# Editorial: Factors affecting performance and recovery in team sports: A multidimensional perspective, Volume II

**DOI:** 10.3389/fphys.2023.1166761

**Published:** 2023-03-09

**Authors:** Athos Trecroci, Damiano Formenti, Jason Moran, Dino Pedreschi, Luca Cavaggioni, Alessio Rossi

**Affiliations:** ^1^ Department of Biomedical Sciences for Health, Università degli Studi di Milano, Milan, Italy; ^2^ Department of Biotechnology and Life Sciences, University of Insubria, Varese, Italy; ^3^ School of Sport, Rehabilitation and Exercise Sciences, University of Essex, Colchester, United Kingdom; ^4^ Department of Computer Science, University of Pisa, Pisa, Italy; ^5^ Department of Endocrine and Metabolic Diseases, Obesity Unit and Laboratory of Nutrition and Obesity Research, IRCCS Istituto Auxologico Italiano, Milan, Italy; ^6^ National Research Council (CNR), Institute of Information Science and Technologies (ISTI), Pisa, Italy

**Keywords:** recovery, fatigue, wellbeing, cognition, perceptual response, soccer, physical performace

The second volume of the Research Topic on Factors Affecting Performance and Recovery in Team Sports continues and extends the knowledge of the first volume (Trecroci, 2022) reinforcing the concept that the analysis of team sports should embrace a multidimensional perspective (i.e., cognitive, physical, technical, physiological, psychological, morphological, and preventive) (Carling et al., 2018; Trecroci et al., 2020a; 2020b; 2021a; 2021b; Formenti et al., 2020). To tackle this challenge, sport science research in team sports is giving importance not only to suitable training strategies and performance monitoring, but also to *ad hoc* recovery and wellness recommendations. By the way, this Research Topic published five experimental studies, and three out of these are on recovery strategies, whereas the other two focus on training methodologies.

The first published manuscript was that of Rossi et al., in which the authors developed a big data analytics framework to predict players’ wellness according to the external and internal workloads performed in the previous days. In this study, 17 elite soccer players competing in the Italian championship (Serie A) filled in a questionnaire about their perceived wellness every morning, and their GPS data and rate of perceived exertion were recorded during and after each training and match. The main finding was that, using a machine learning framework, players’ perceived wellness was accurately estimated by workload history as input, thus demonstrating the usefulness of machine learning approaches in the monitoring process of training and recovery in team sports athletes. This would improve the decision-making process of the coaching staff in scheduling the training workloads in each phase of the season.

The study by Pernigoni et al. focused on the recovery of basketball players, and aimed: i) to assess the perceived usefulness and actual use of recovery strategies in basketball practitioners; ii) to identify potential barriers that may prevent the use of recovery strategies in basketball. The analysis of the survey completed by 107 practitioners (contacted by e-mail, phone, and social media) revealed that a dissociation between scientific evidence and perceived effectiveness exists. Regarding actual use, participants tended to favor easily implementable strategies (e.g., active recovery, stretching), rather than evidence-supported expensive and/or impractical interventions (e.g., whole-body cryotherapy). It is evident the need of research on practical low-cost solution to be applicable in a real-world setting.

Recovery was also the topic of the study by Rebello et al. and focused on sleep, fundamental for the attainment, maintenance, and restoration of physical and mental health. In this brief research report, the Authors investigated the sleep quality and sleep behaviors in Canadian university collegiate athletes using validated questionnaires tailored specifically for athletes. It was shown that sleep quantity, quality, and behaviors were suboptimal for many athletes. Moreover, half of the university athletes did not meet the thresholds for adequate sleep, and some of them could meet clinical sleep issues. Although preliminary, these findings provide a clear picture of scarce sleep hygiene in university collegiate athletes, showing the need for strategies to promote optimal sleep hygiene, such as educational workshops.

A brief research report by Ojeda-Aravena et al. focused on an experimental study on plyometric training. The study aimed to compare jump-related performance after plyometric training on harder *versus* softer surfaces in rugby sevens’ players using a quasi-experimental randomized parallel design. Fourteen players were randomly assigned to the harder surface group and softer surface. Before and after the 4-week intervention period, performance on squat jump, countermovement jump, and countermovement jump with arms was assessed. Countermovement jump with arms on softer surfaces improved vertical jump displacement and lower body power after 4 weeks of plyometric training, demonstrating its superiority against harder surfaces for improving jump and power in sevens rugby players.

Also, the study by Hammami et al. focused on plyometric training. Although previous research in pediatric populations has revealed performance enhancements following long-term plyometric training, the acute effects of plyometric exercises on performance-related variables are still unclear. This study tried to fill this gap by investigating the acute effects of maximal vs. submaximal hurdle jump exercise protocols executed during one training session on balance, vertical jump, reactive strength, and leg stiffness in young volleyball players. Thirty male young volleyball players randomly performed a maximal and submaximal hurdle jump exercise protocols. While the maximal protocol was more effective to induce acute performance improvements in balance, reactive strength index, and vertical jump, the submaximal protocol induced greater improvement on leg stiffness. As performance in volleyball requires both postural control and muscle strength/power, these results suggest that young volleyball players should implement dynamic plyometric protocols involving both maximal and submaximal hurdle jump exercises during warm-up, thus improving subsequent balance performance and muscle strength/power.

We believe that the articles of this Research Topic provide an exciting overview of the current state of research on the factors affecting performance and recovery in team sports, highlighting the varsity of variables that can play a role in the whole training process. We hope that they will stimulate further studies to advance knowledge in team sports science with a rigorous scientific approach, but also with high applicability to the real-world setting. It is worth keeping in mind that sports science research should try to give answers to practitioners in the field as part of a multidimensional theme. For example, future research will have to assess the effects of different training process and recovery strategies on the long term. However, scientific evidence-based approaches are not often easily accessible by the majority of practitioners. It is such an issue that should be the focus of further research in sport and exercise science, i.e., the applicability and feasibility of evidence-based solutions to the real word setting.
